# Differences in women’s experiences of labour according to type of fetal monitoring: a quantitative analysis of an Australian national survey

**DOI:** 10.1186/s12884-025-07509-z

**Published:** 2025-05-13

**Authors:** Kate M. Levett, Deborah Fox, Panashe Bamhare, Rebecca Coddington, Kerry L. Sutcliffe, Elizabeth Newnham, Vanessa Scarf

**Affiliations:** 1 School of Medicine, The University of Notre Dame Australia, Sydney, Australia; 2 Collective for Midwifery, Child and Family Health, University of Technology Sydney, Sydney, Australia; 3 NICM Health Research Institute, Western Sydney University, Sydney, Australia; 4 Translational Health Research Institute, Western Sydney University, Sydney, Australia; 5 Western Sydney Area Health Service, Westmead Hospital, Sydney, Australia; 6 School of Nursing and Midwifery, Flinders University, Adelaide, Australia; 7 School of Nursing and Midwifery, Newcastle University, Newcastle, Australia; 8 Waminda – South Coast Women’s Health & Welfare Aboriginal Corporation, Nowra, Australia

**Keywords:** Fetal monitoring, Electronic fetal monitoring, Antenatal education, Maternal health, Maternity care, Maternal experience

## Abstract

**Intro:**

While monitoring of the fetal heart rate in labour is recommended, few studies have compared women’s experiences of different forms of monitoring technologies, their impact on labour and perceived risks and benefits.

**Methods:**

The Women’s experiences of Monitoring Baby (WOMB) study, an Australian national survey, examined experiences of intrapartum fetal monitoring in labour. This study is one of two quantitative analyses of survey responses received.

**Results:**

We received 861 valid responses. The most common form of monitoring across all hospital settings was wired CTG (53% of total). Women who used wired CTG were more likely to be primiparous (OR = 3.220, [95%CI:2.080–4.987], *p* < 0.001), and give birth at a private hospital (OR = 3.017 [95%CI:1.632–5.576], *p* < 0.001). Women who were monitored via wired CTG were more likely to use pharmacological pain management, and have an emergency caesarean section (*p* < 0.001), which remained significant when adjusting for epidural. Women who gave birth vaginally were more likely to have been monitored via intermittent auscultation (OR = 3.582, [95%CI:2.007–6.390], *p* < 0.001), and to use non-pharmacological techniques such as mobility (*p* < 0.001) and supportive care (*p* < 0.01). Of the women monitored via wired CTG 58% felt that monitoring had a negative impact on their labour.

**Conclusion:**

This study has substantial implications for research, policy and practice, including the implementation of less invasive and more humanised forms of fetal monitoring. The promotion of freedom of movement and bodily autonomy in labour is essential. This includes implementation of evidence-based practices and information about methods of fetal monitoring that support woman-centred care and optimise physiological processes.

## Background

Over 300,000 women give birth in Australia each year, with intrapartum monitoring of the fetus being recommended on either an intermittent or continuous basis [1, 2]. Intermittent monitoring with handheld Doppler or Pinard’s stethoscope is indicated for women whose pregnancies are considered low risk and labour is progressing normally. Where pregnancies are complex, or risk factors develop during labour, then continuous monitoring is recommended. In Australia, it is estimated that more than 50% of women experience continuous electronic fetal monitoring (EFM), commonly via cardiotocography (CTG) during their labour [2, 3], despite a relative lack of evidence of benefit [4, 5]. Rates of interventions such as induction and augmentation of labour are rapidly increasing, particularly for primiparous women [3], which is necessitating an escalation of the use of continuous electronic fetal monitoring, impacting women’s experience of labour, midwifery skills and confidence, as well as broader systemic issues with hospital systems [6, 7].

Monitoring during labour can impact women both psychologically and physiologically, affecting their ability to be upright and mobile in labour and restricting their sense of bodily autonomy [8]. Mobility during labour has been shown to decrease length of labour, and reduce rates of epidurals and the likelihood of caesarean sections (CS), with no negative impact for women and babies [9, 10]. Mobility during labour also improves women’s perception of control and choice in labour [10, 11], which decreases levels of stress and pain, and the need for pharmacological pain management [12]. It is therefore essential that options for fetal monitoring optimise humanised care by enhancing bodily freedom and agency during labour [8].

Telemetry options for EFM which are wireless devices, have been available in Australia since 2003, but there has been limited uptake, with Australian survey research indicating that most hospital respondents had fewer than five machines regardless of size of institution [8]. This is despite findings that wireless monitoring has a positive influence on women’s freedom of movement and sense of control and choice in labour [8]. Research from the United Kingdom (UK) suggests that uptake has been higher, with 62% of responding hospitals reporting that they have telemetry monitoring devices [13]. However, despite hospitals having wireless EFM technology, many maternity units reported having fewer than three machines, suggesting that women are still mostly monitored using wired CTG. This research also suggests that workplace culture and models of care are part of the issue that while newer technology which promotes movement and comfort are available, they are not promoted for routine use [13]. To date, research has noted women’s experiences of having fetal monitoring [14], however there has been limited research that has examined women’s experiences of fetal monitoring during labour comparing different types of monitoring used.

This study is one of two papers which reports on the quantitative results from the larger WOMB Study, a national survey of women about their experiences of fetal monitoring in labour in Australia. This paper examined differences in women’s experiences of labour according to type of fetal monitoring, and its association with parity, place of birth, pain management used, mode of birth and perception of benefit for themselves and their babies.

## Aims and objectives

The WOMB Study aimed to describe women’s experiences of intrapartum fetal monitoring in an Australian national survey. This paper examined women’s survey responses about their experiences according to method of fetal monitoring they received in the intrapartum period.

Therefore, we aimed to:

1. Evaluate differences in experiences of labour and birth according to type of fetal monitoring received.
2. Evaluate differences in fetal monitoring type according to women’s parity and place of birth.
3. Evaluate differences according to type of fetal monitoring received and type of pain management used in labour and mode of birth.
4. Understand if women felt intrapartum fetal monitoring was beneficial for themselves and their baby, and if women felt monitoring had a negative impact on themselves or their baby.

## Methods

### Study design

This study was a national cross-sectional survey which explored experiences of intrapartum fetal monitoring in Australia. Data were collected from 30th May to 30th June 2022. Quantitative and text response qualitative data were collected via an online survey developed by the authors using Qualtrics^®^ software [15]. Women self-selected into the survey, and were eligible to participate if they were able to read English, were over 18 years of age, had given birth in the previous five years to one or more babies in Australia, and they had some form of fetal monitoring during their labour. Pilot testing of the survey was conducted with a number of stakeholders, including researchers, clinicians and women who had experienced monitoring. The survey was revised according to feedback prior to project commencement.

This paper is one of two that report on quantitative responses to the WOMB study survey. A qualitative content analysis of open-ended responses to survey questions has been reported elsewhere [16].

### Survey design

This national survey study sought consumer input to inform implementation of planned future research. An advisory group (steering committee) was formed to guide the proposed study questions, inform future planned clinical trials investigating optimal application of fetal monitoring, and the translation of findings for guidelines and government policy. The advisory group, which included researchers, clinicians, knowledge translation experts and community members, including women with lived experience, met regularly and participated in implementation strategies for recruitment to ensure as broad a population as possible, and provided expert project advice. The design of the survey was based on the expertise of the research team members. Pilot testing was conducted with 10 consumers and stakeholders prior to project commencement. The final survey version was distributed via Qualtrics^®^ software [17] using an online link. The survey ascertained demographic data, as well type of hospital attended, participation in childbirth and parenting education classes, what sources were used to obtain information about monitoring, depth of discussion with care providers regarding monitoring, what type of monitoring was used, what pain management strategies were used, mode of birth, and what were women’s experiences of monitoring during labour and birth. The quantitative outcomes are reported here and in a separate paper [18]. Text responses were used to gather further information or for clarification where required, and are reported in a content analysis separately [16].

### Recruitment

The survey was distributed online via a Qualtrics link, which was shared broadly across Australia using various social media platforms and distribution networks to ensure as broad a population as possible. It was distributed on multiple parenting websites, including paid advertisements through the ‘Mum’s Network’ and distributed via Facebook. The authors also distributed the survey through their own professional networks and online social media.

### Ethics

Ethics approval was received from the University of Technology Sydney’s Human Research Ethics Committee (approval no. ETH21-6563) and the University of Notre Dame Human Research Ethics Committee (approval no. 2022–063 S) and adhered to the Declaration of Helsinki Ethical Principles for Medical Research Involving Human Participants. At the start of the survey, a participant information section was provided, and consent was sought via a click button enabling access to the survey. Participants were able to discontinue the survey at any time and anonymous data was submitted upon completion. In case of any distress from participation, helpline information was provided at the beginning of the survey.

### Data analysis

We used the Statistical Package for the Social Sciences (SPSS) version 23 [19] to complete the analysis of quantitative data. The data were de-identified, cleaned and coded in Excel prior to SPSS. Descriptive statistics performed included counts and percentages. Continuous variables used student t-tests, and categorical variable analysis was performed using Chi squared tests. Missing data is indicated and accounted for in the analysis, as response rates varied between questions.

### Categories for monitoring type

For analysis of associations between type of monitoring and outcomes presented in this paper, the type of monitoring was categorised according to the main type of monitoring experienced during labour as identified by the respondent (excluding the admission CTG if performed). If ‘multiple’ forms of monitoring were identified by the respondent, where more than one primary type of monitoring experienced during labour, we categorised this as ‘multiple monitoring’. They were categorised as;

1. Handheld monitoring (Pinards and doppler),
2. Wireless CTG monitoring (telemetry with belts, no wires),
3. Wired CTG monitoring (transducers with belts and wires),
4. NIFECG (non-invasive electrocardiography/adhesive electrode monitoring (beltless and wireless),
5. Fetal scalp electrodes (attached directly to fetal scalp), or.
6. Multiple monitoring, if the respondent indicated that they had more than one form of primary monitoring type other than an admission CTG.

## Results

There were 861 women who responded to this survey from every Australian state and territory (Table 1), with 798 responses indicating type of monitoring received. Any missing data for individual variables are reported for each variable. Respondents’ average age was 33 years (*±* 5.2 years), and the majority of respondents were Australian born (85.5%), primiparous (62.9%), had tertiary level education (71.7%), and were married or in de-facto relationship (95%). There were 28 (3.3%) of respondents who identified as Aboriginal or Torres Strait Islander [3]. In this sample of women, the most common birth setting was a local public hospital in a rural or remote location (30.2%) or a local public hospital in a metropolitan area (24.8%).

**Table 1:** Respondent characteristics

Characteristics	*n* = 861 (missing = 30 (3.4%))	(%)
**Age**	Mean = 33.0 y (*±* 5.2)	
**Education**: (n = 861)		
<Yr 12 equiv	14	1.6
Yr12 equiv	55	6.4
TAFE	153	17.7
Bachelors	321	37.3
Postgraduate	318	36.9
**Country of birth**		
Australia	762	88.5
NZ/UK/USA/Canada/Europe	86	10.0
Asia	13	1.5
**Identify as Aboriginal and/or Torres Strait Islander**		
Yes	28	3.3
**Parity**		
Primiparous	560	65.0
Multiparous	298	34.6
Missing	3	0.4
**Birth Location**		
Large/Tertiary Hospital	141	16.4
Local Public Hospital City	221	25.7
Private Hospital City	87	10.1
Birth Centre Hospital (alongside)	53	6.2
Birth Centre freestanding	1	0.1
Public Hospital Rural Remote	269	31.2
Private Hospital Rural Remote	62	7.2
Missing	27	3.1
**State**		
NSW	268	31.1
ACT	102	11.9
VIC	170	19.7
QLD	165	19.2
SA	31	3.6
WA	51	5.9
TAS	28	3.3
NT	34	3.9
Missing	12	1.4
**Relationship status**		
Married	614	71.3
De-facto	204	23.8
Separated	15	1.7
Single	21	2.5
Divorced	2	0.2
Other	5	0.5
Missing	0	0.0

### Monitoring type and information provided

The 798 respondents experienced 1364 individual types of monitoring, as some women had more than one type applied during labour. Women reported having their babies monitored during the admission process (admission CTG) (*n* = 199), and during labour (*n* = 798). Of the total population, 58.7% (*n* = 335) reported having multiple monitoring types with an average of 1.5 forms of monitoring for each woman, however many women had three to four types of monitoring recorded for their labour and birth, which overall gave a total sample of 1364 records of monitoring (Table 2).

Excluding the admission CTG, monitoring during labour was undertaken via a variety of devices. Overall, there were 1,165 episodes of monitoring used by 798 women who completed the survey. Of the total episodes of monitoring (*n* = 1,165), these included (1) handheld devices (Pinards and doppler) (*n* = 229, 19.7%), (2) Wireless/telemetry (transducers with belts) (*n* = 276, 23.7%), (3) Wired (transducers with belts and wires) (*n* = 423, 36.3%), (4) NIFECG adhesive electrodes (beltless and wireless) (*n* = 10, 0.9%), (5) Fetal scalp electrode (attached to the baby’s head) (*n* = 100, 8.6%) and (6) ‘Other’ (*n* = 127, 10.9%), where women generally indicated a combination of monitoring, without specifying any type. Of the 798 survey responses, the most common type of monitoring that women experienced was wired CTG monitoring (*n* = 423, 53%), and the least common was adhesive electrode monitoring (*n* = 10, 1.3%).

**Fig. 1:**
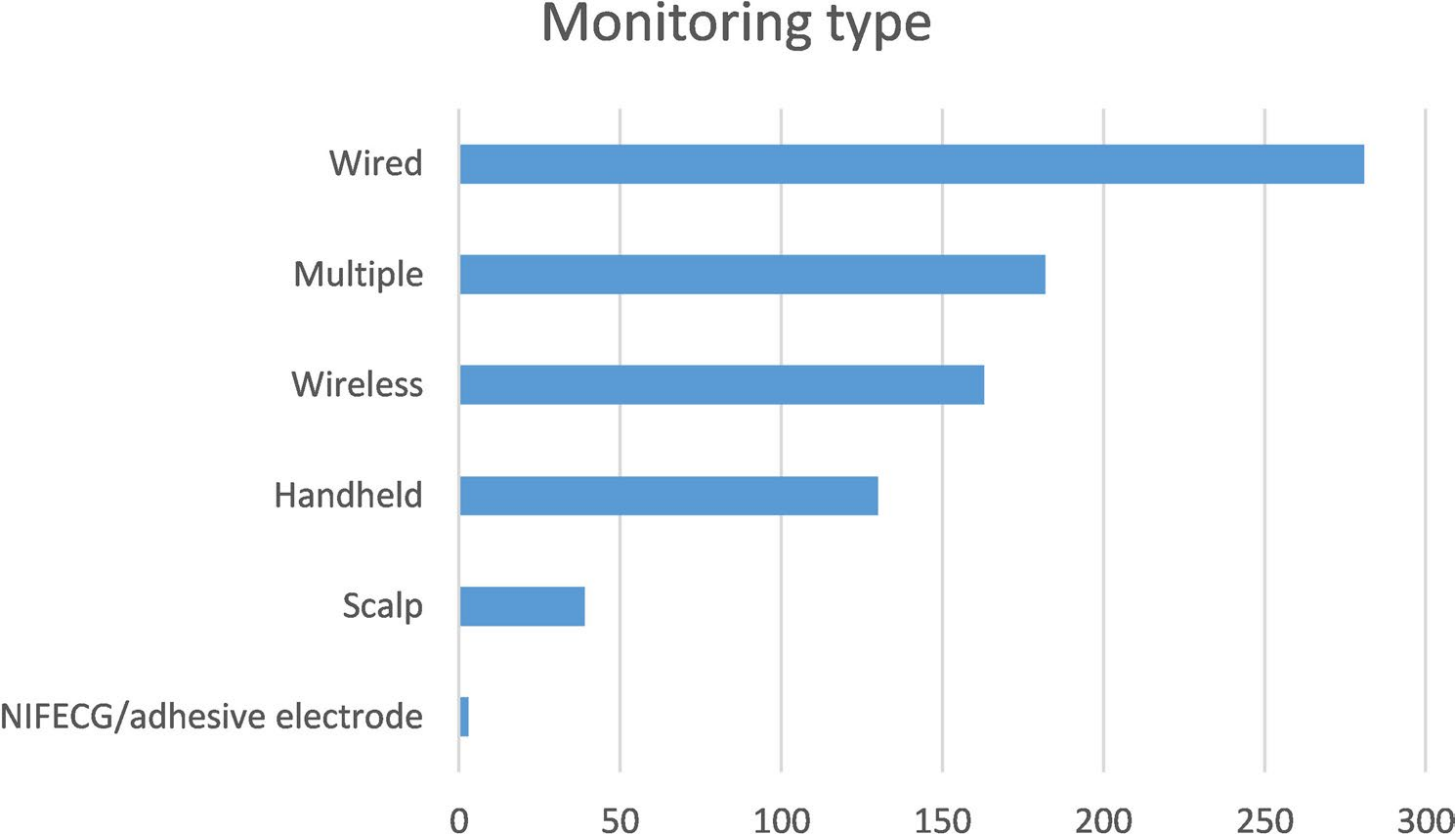
Frequency of monitoring types

For analysis of associations between type of monitoring during labour and outcomes, women were categorised as to the main type of monitoring experienced during their labour. Where there was more than one main type of monitoring used in a single labour, respondents were categorised as having ‘multiple’ monitoring (182, 22.8%) (see Fig. 1).

### Analytic statistics

We analysed type of monitoring used and its association to outcomes from the survey. For this analysis, we used the categorisation according to primary type of monitoring as described above. Additionally, we performed binary and multivariate logistic regression analyses for monitoring type (using handheld monitoring as the reference category), with selected outcomes (mode of birth, public/private, pain management strategies) to examine individual associations. A Bonferroni correction was used due to multiple dependent variables for pain management analysis (*n* = 11). We report the odds ratios and 95% confidence intervals (OR= [95%CI], p value).

### Confounding variables

To control for confounding variables we considered parity, epidural and induction of labour as potential confounding factors for mode of birth. We stratified for parity (primiparous and whole of group) and for epidural (no epidural and whole of group) in separate analyses. However, we were unable to establish valid data for the outcome of induction of labour, and therefore did not include it in this analysis. Induction of labour is a common indication for any type of continuous EFM, and this was noted in text responses from women. However, this study focused on women’s experiences of different forms of monitoring regardless of onset of labour. Results should be considered in this light.

### Monitoring type according to parity

For analysis of outcomes, we categorised women’s parity as either primiparous (if this was their first birth > 20 weeks’ gestation) or multiparous (if they had one or more previous births > 20 weeks’ gestation). The analysis showed that primiparous women were significantly more likely to have continuous electronic fetal monitoring via either wired or wireless CTG, and multiparous women were more likely to have intermittent auscultation via handheld doppler during labour.

**Table 2:** Primary monitoring according to parity

Monitoring type	First Baby (Primigravida)			p value
	**Yes (%)**	**No (%)**	**Total**	
Handheld Doppler	61 (11.7)	68 (52.7)	129	*p* < 0.001**
Wireless CTG	104 (20.0)	59 (36.1)	163	
Wired CTG	208 (40.1)	72 (25.7)	280	
Fetal scalp electrode	22 (4.2)	17 (43.6)	39	
NIFECG/adhesive electrodes	2 (66.6)	1 (33.3)	3	
Multiple	122 (67.4)	59 (32.6)	181	
Total	**519**	**276**	**795**	

To further explore the association between parity and monitoring type, we conducted a multinomial logistic regression model. After adjusting for age, women who had wired monitoring, versus handheld, were more than twice as likely to be primiparous (OR = 3.220 [95%CI:20.181–4.987], *p* < 0.001). Women who had multiple forms of monitoring, compared to handheld, were also more than twice as likely to be primiparous (OR = 2.305 [95% CI:1.448–3.669], *p* < 0.001).

### Monitoring type and place of birth

When examining differences for monitoring type according to place of birth, we found that there were significant differences in monitoring types used at different places of birth (*p* < 0.001). For this data, ‘Birth Centres’ which are midwifery-led units, were either ‘alongside’ a hospital unit (AMU) or ‘freestanding’ (FMU), and were categorised together due to low numbers in the freestanding category. Wired CTG monitoring was the most common form of monitoring used across most hospital settings. While wired CTG monitoring constituted 37% of monitoring overall, it was used at rates of nearly 63% in private hospitals in metropolitan areas, nearly 42% in private hospitals in rural or remote areas, and nearly 40% at public hospitals in metropolitan areas. The next most common form of monitoring was via multiple forms of monitoring, which was used on average for 24% of women. Usually this resulted from wireless CTG progressing to wired CTG and/or fetal scalp electrodes. This was most commonly found in public hospitals in both metropolitan, and rural/remote areas.

Handheld dopplers were used most frequently at birth centres and in home birth settings, wireless telemetry devices were used most frequently at private hospitals in rural/remote settings (24.1%), and wired CTG monitoring was most frequently used at private hospitals in metropolitan areas (62.7%) (Table 3).

**Table 3:** Monitoring according to place of birth

Monitoring Type				Place of birth - all women						
	**TOTAL** **N (%)**	Public Large/ Tertiary Hospital n (%)	Public Local Hospital Metro n (%)		Public Birth Centre (hospital) n (%)	Public Hospital rural remote n (%)	Private Hospital Metro n (%)	Private Hospital rural remote n (%)	Home birth (public or private) n (%)	p value
Handheld	130 (16.3)	23 (17.8)	16 (8.0)		24 (48)	33 (13.1)	9 (10.8)	5 (8.6)	20 (95)	< 0.001
Wireless	161 (20.3)	23 (17.8)	43 (21.4)		7 (14)	57 (22.6)	16 (19.3)	14 (24.1)	1 (5)	
Wired	281 (35.2)	43 (33.3)	73 (36.3)		9 (18)	81 (32.1)	52 (62.7)	23 (39.8)	0	
Fetal scalp	37 (5.0)	5 (4.0)	15 (7.5)		0	13 (5.2)	1 (1.2)	3 (5.1)	0	
NIFECG	3 (0.4)	0	2 (1.0)		0	1 (0.4)	0	0	0	
Multiple	182 (22.8)	35 (27.1)	52 (25.8)		10 (20)	67 (26.6)	5 (6.0)	13 (22.4)	0	
TOTAL	794 **(100%)**	129 **(100%)**	201 **(100%)**		50 **(100%)**	252 **(100%)**	83 **(100%)**	58 **(100%)**	21 **(100%)**	

To further explore the associations between public and private hospital types and monitoring, we conducted a multinomial logistic regression model. After adjusting for age, women who gave birth in a private hospital were more than 3 times as likely to have wired monitoring compared to handheld, than if they gave birth in a public hospital (OR = 3.115 [95% CI: 1.680–5.780], *p* < 0.001).

### Monitoring type and place of birth: primiparous women

We analysed monitoring type associated with parity and place of birth, and found that for primiparous women, handheld monitoring was most commonly used in birth centres and homebirth settings, but the rate of use was less than overall rates including multiparous women. Overall, 40% of women received wired CTG monitoring, with this form of monitoring being the most common for both private and public hospitals. The largest proportion of primiparous women being monitored via wired CTG monitoring occurred at private hospital settings in metropolitan areas (67%), and private hospitals settings in rural or remote areas (42.5%). Overall, the difference remained significant for this population (*p* < 0.001) (Table 4).

**Table 4:** Monitoring according to place of birth for primiparous women

Monitoring Type			Place of birth - primiparous women							
	TOTAL N (%)	Public Large/ Tertiary Hospital n (%)	Public Local Hospital Metro n (%)	Public Birth Centre (hospital) n (%)	Public Hospital rural remote n (%)	Private Hospital Metro n (%)	Private Hospital rural remote n (%)	Home birth (public or private) n (%)	p value	
Handheld	61 (11.8)	9 (10.7)	9 (6.6)	14 (46.7)	16 (9.9)	6 (10.5)	1 (2.5)	6 (85.7)	< 0.001	
Wireless	103 (20.1)	16 (19.0)	23 (16.9)	4 (13.3)	36 (22.2)	10 (17.5)	13 (32.5)	1 (14.3)		
Wired	208 (40.1)	31 (36.9)	57 (41.9)	6 (20)	59 (36.5)	38 (66.7)	17 (42.5)	0		
Fetal scalp	20 (4.2)	2 (2.4)	8 (5.9)	0	8 (4.9)	0	2 (5.0)	0		
NIFECG	2 (0.3)	0	1 (0.7)	0	1 (0.6)	0	0	0		
Multiple	122 (23.5)	26 (31.0)	38 (28.0)	6 (20)	42 (25.9)	3 (5.3)	7 (17.5)	0		
TOTAL	516 **(100%)**	84 (100%)	136 **(100%)**	30 **(100%)**	162 **(100%)**	57 **(100%)**	40 **(100%)**	7 **(100%)**		

To further explore the associations between public and private hospital types and monitoring for primiparous women, we conducted a multinomial logistic regression model. After adjusting for age, primiparous women who gave birth in a private hospital were more than three times as likely to have wired monitoring, compared to handheld, than primiparous women who gave birth in a public hospital (OR = 3.378, [95% CI: 2.123–5.405], *p* < 0.001).

### Mode of birth and fetal monitoring type

We examined the association between intrapartum monitoring type and mode of birth. Women who had wired monitoring had an increased likelihood of having an emergency caesarean section (emCS) (OR = 3.582, [95%CI: 2.007–6.390], *p* < 0.001). Monitoring with handheld, wireless, fetal scalp electrodes, or multiple forms of monitoring, was associated with increased likelihood of a normal vaginal birth (including for women with previous caesarean and vaginal breech births) (*p* < 0.001). To control for epidural as a potential confounding factor for emCS, we excluded women with epidural and analysed the data for only women who did not have an epidural (*n* = 447). For women with no epidural, wired monitoring remained significantly associated with an increased likelihood of having an emCS (OR = 2.823 [95%CI: 1.741–4.576], *p* < 0.001) (Table 5).

There were insufficient data to examine any association for NIFECG monitoring and mode of birth.

**Table 5:** Monitoring type and mode of birth

	Mode of birth				
Monitoring Type	NVB	Instrumental VB	EmCS	Total	***X***^**2**^**(DF)**, **p value**
Handheld *n* = 117	94 (80.3)	11 (9.4)	12 (10.3)	117	< 0.001
Wireless *n* = 158	98 (62.0)	26 (16.5)	34 (21.5)	158	
Wired 270	106 (39.3)	73 (27.0)	91 (33.7)	270	
Fetal scalp *n* = 35	26 (74.3)	6 (17.1)	3 (8.6)	35	
NIFECG *n* = 3	2 (66.7)	1 (33.3)	0 (0.0)	3	
Multiple *n* = 179	82 (45.8)	44 (24.6)	53 (29.6)	179	
Total	**408 (53.5)**	**161 (21.1)**	**193 (25.3)**	**762**	**78.7 (15)**

To further examine the associations between mode of birth and monitoring for women, we conducted a multinomial logistic regression model. After adjusting for age and epidural use, women who had a caesarean section were more than three times as likely to have wired monitoring, compared to handheld (OR = 3.582, [95%CI: 2.007–6.390], *p* < 0.001. However, when we adjusted for parity in the model, there was no longer a significant association for monitoring and mode of birth, suggesting that first baby is a moderating factor in mode of birth.

**Fig. 2:**
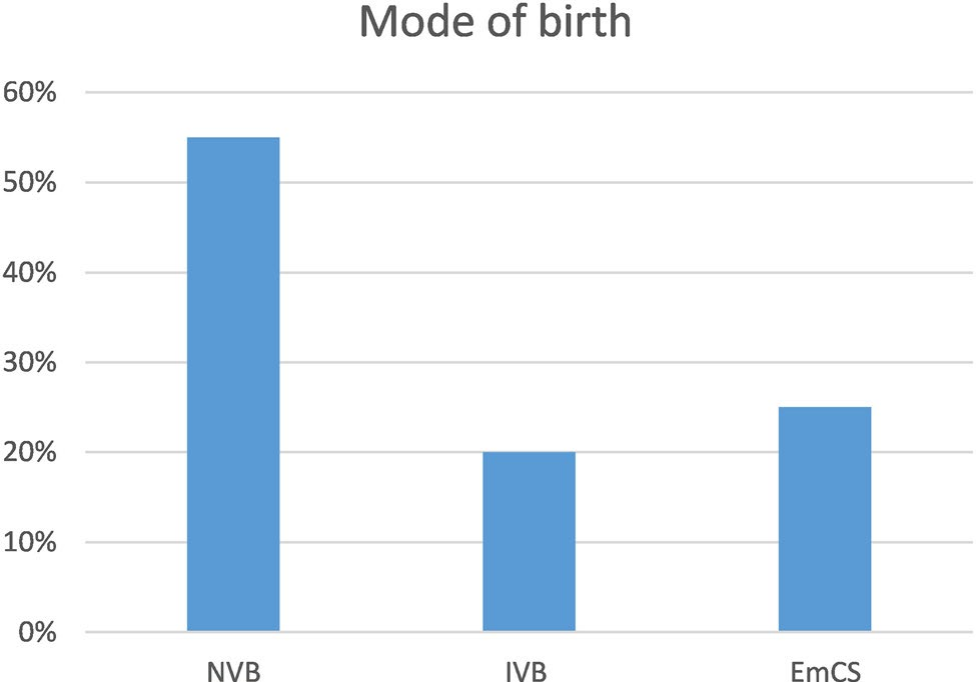
Mode of birth

Overall, regardless of monitoring type, most women had a normal vaginal birth (NVB) (53.5%), and 25% of women had an unplanned (emergency) caesarean section (emCS) (see Fig. 2).

### Pain management techniques used

We examined women’s reported use of pain management, according to the primary type of monitoring used in their labour, using an individual chi squared analysis. Women who were being monitored intermittently via handheld doppler were more likely to use non-pharmacological pain relief techniques such as, breathing (*p* < 0.001), water/shower, bath (*p* < 0.001), manual therapies (acupressure/ massage/ yoga) (*p* < 0.001), movement (*p* < 0.001) and supportive care (emotional/physical comfort measures) (*p* < 0.001). Women who were being monitored via wireless CTG were more likely to use breathing techniques (*p* < 0.01), movement (*p* < 0.001) and supportive care from a partner or care provider (*p* < 0.01).

Women who were being monitored via wired CTG were more likely to use pharmacological pain management, including nitrous oxide gas (*p* < 0.01) and epidural analgesia/anaesthesia (*p* < 0.001). Women who were monitored via a fetal scalp electrode were likely to use breathing techniques (*p* < 0.001) and supportive care (*p* < 0.001). There was insufficient data for women who used the NIFECG monitoring for analysis.

**Table 6:**
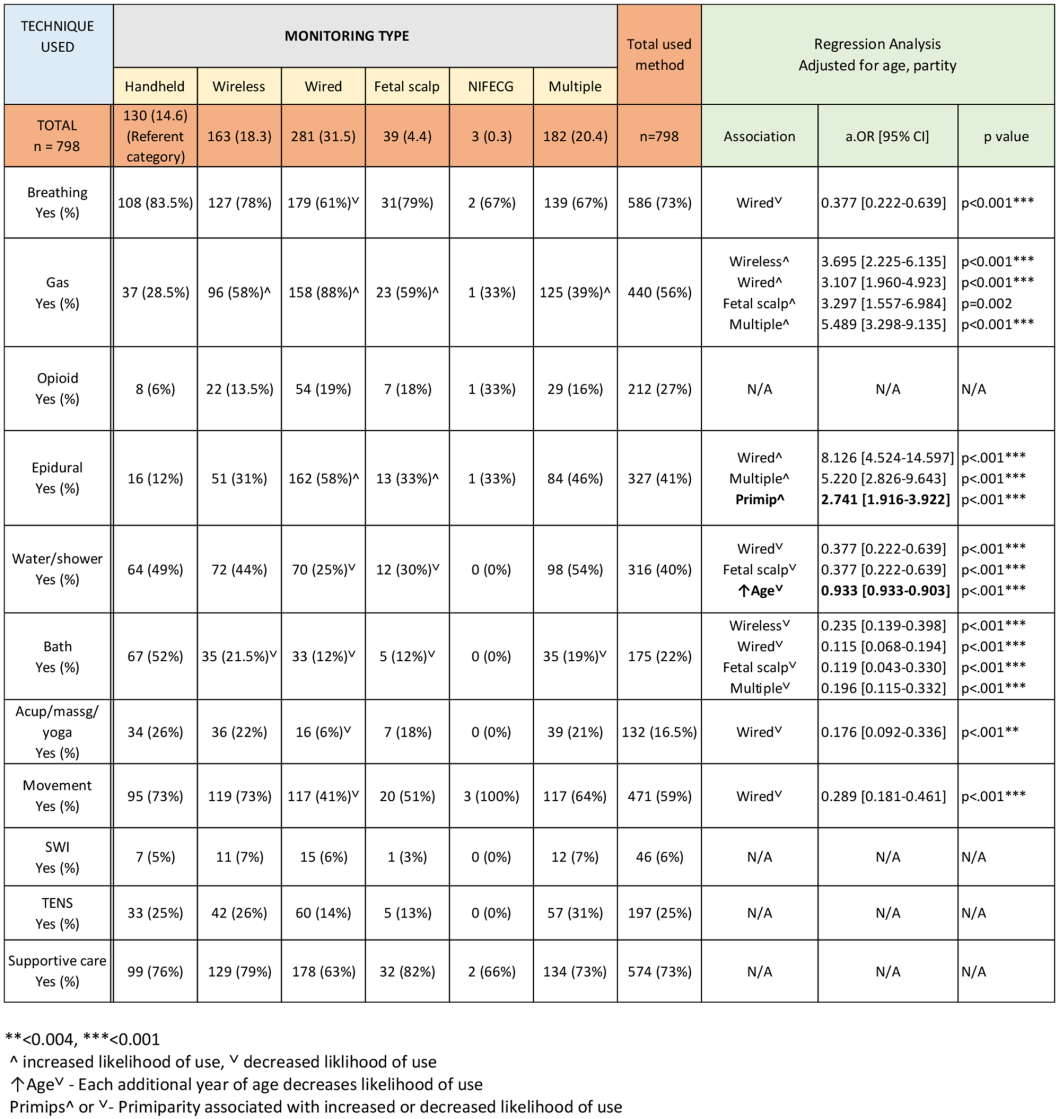
Pain management techniques used according to monitoring type

To examine individual associations, we used binomial regression analyse modelling for each monitoring type, to examine individual associations and determine odds ratios for use of pain managements strategies. Adjusting for parity and age, we used the lowest risk categorisation of handheld monitoring as the referent category and compared to all other monitoring categories. Individual associations and likelihood of use is shown in Table 6, with decreased likelihood of use indicated by ^∨^ and increased likelihood of use is indicated by **^.** Associations with adjusted age and parity are indicated in the final column.

The results from the binomial regression analysis for wired monitoring, adjusted for age and parity, show that women who had wired monitoring, compared to women who were monitoring via handheld devices, were less likely to use breathing techniques OR = 0.377 [95%CI: 0.222–0.639] *p* < 0.001), the bath (OR = 0.115 [95% CI: 0.068–0.194] *p* < 0.001), acupressure/massage (OR = 0.176 [95% CI: 0.092–0.336] *p* < 0.001), or movement (OR = 0.289 [95% CI: 0.181–0.461] *p* < 0.001). However, they were more likely to have used N02 gas (OR = 3.107 [95%CI: 1.960–4.923] *p* < 0.001), and to have used epidural analgesia (OR = 8.126 [95%CI: 4.524–14.597] *p* < 0.001), which was also significantly associated with primiparity. Outcomes for other pain management strategies and types of monitoring are indicated in Table 6.

### Perception of benefit of fetal monitoring for themselves and their baby

We asked women if they felt reassured by the type of fetal monitoring they received during labour. In a series of statements, we asked; (1) if women felt that monitoring had a ***beneficial*** impact on themselves or their babies, and (2) if women felt that fetal monitoring had a ***negative*** impact on themselves or their babies during labour.

**Fig. 3:**
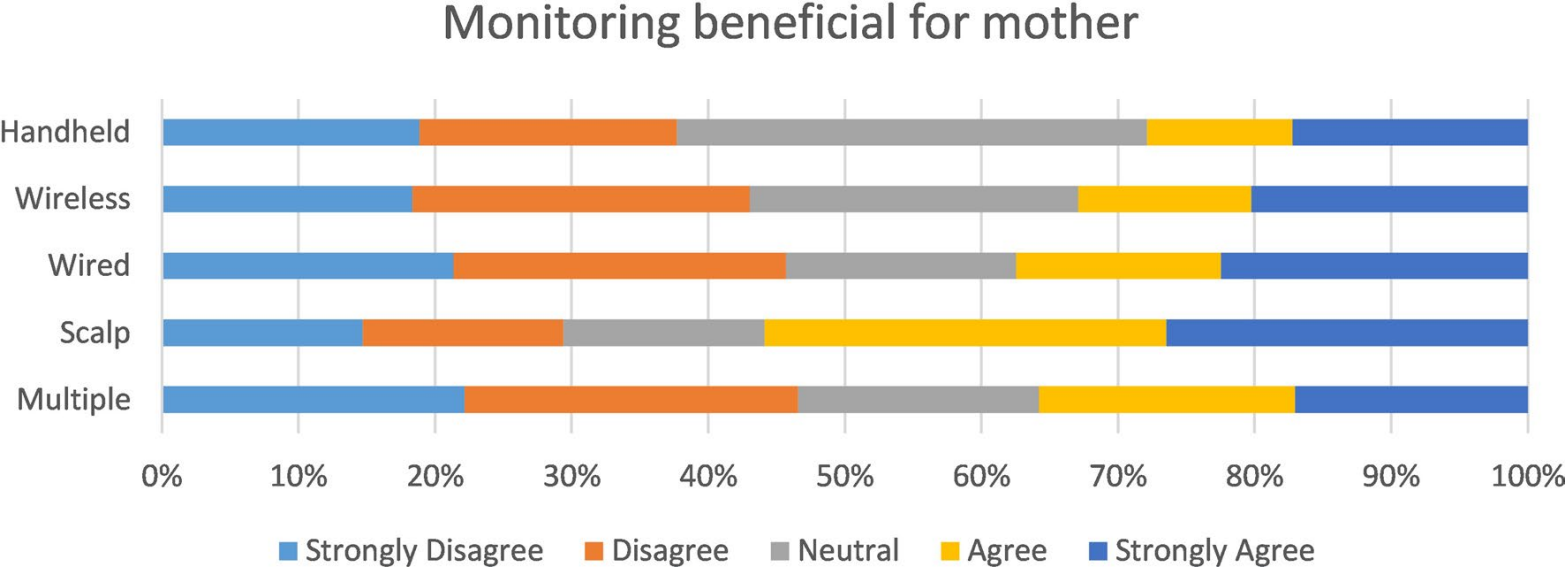
Perceived benefit of monitoring for mother

Women who were monitored via wired CTG were more likely to ‘agree’ or ‘strongly agree’ with the statement that monitoring had a negative impact on them during labour (*X*^2^ = 61.8, *p* < 0.001) (Fig. 3).

**Fig. 4:**
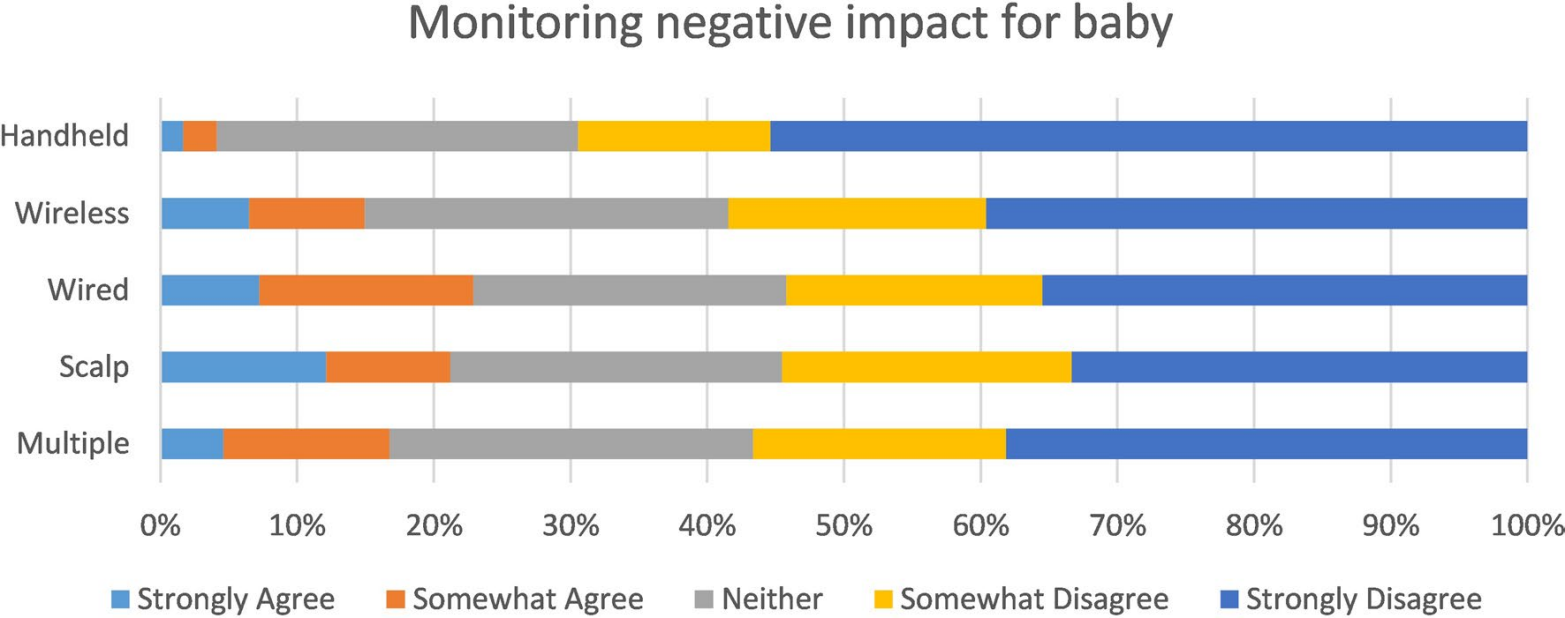
Perceived impact for baby

However, regardless of the type of monitoring received, the majority ‘disagreed’ or ‘strongly disagreed’ that monitoring had a negative impact on their baby (*X*^2^ = 36.4, *p* < 0.01) (Fig. 4).

## Discussion

This study examined 861 valid responses to a 2022 Australian national survey which asked women and birthing people about their experiences of fetal monitoring during labour. We examined if there were differences in experience according to monitoring types, parity, hospital type, type of pain management utilised, and perception of benefit or harm for themselves and their babies. We found that place of birth was significantly associated with type of monitoring received, and that there were significant differences in women’s experiences of labour, and their use of pain management, depending on type of monitoring used, as well as parity. The perceived impact of monitoring as positive or negative was significantly different between monitoring types, especially when women received wired monitoring. In this study the highest proportion of women received continuous wired CTG monitoring, with highest use in private hospital settings and more commonly for primiparous women. Fewer than 20% of women received intermittent monitoring with handheld devices, which was more likely in Birth Centre (FMU/AMU) settings, which may indicate use according to low-risk women as well as workplace culture. Currently in Australia more than 50% of women receive continuous EFM in labour, without evidence of benefit [4]. This apparent escalating use of EFM on women, especially primiparous women, who are at low risk of complications is occurring despite evidence of its negative impact on women’s experience, trajectory of care and the erosion of clinical skills and confidence to support women at low risk of complications [6, 7, 20, 21]. This study has substantial implications for hospital resources and guidelines pertaining to fetal monitoring, as well as implementation of practices that support woman-centred models of care.

### Type of monitoring used

We found that wired CTG monitoring was the most common form of monitoring with 35% of respondents overall, and 40% of primiparous women, stating this was their primary form of monitoring. This was consistent across most places of birth, except private hospitals in metropolitan areas, where over 60% of all women, and 67% of primiparous women experienced wired CTG monitoring. Wired CTG monitoring is ubiquitous in Australian hospitals [8], which is also reflected in our survey. Despite widespread use of continuous CTG monitoring, the use of telemetry – a wireless form of continuous monitoring designed to enable greater freedom of movement, remains limited [8]. Despite wireless monitoring technology having been available for decades, many hospitals still do not have this routinely available, hence wired CTG monitoring still predominates in many settings [8, 13]. This has implications for women at all levels of risk, particularly for primiparous women, as they were more likely to receive wired monitoring in this study. Given that wired monitoring was more likely to be associated with increased incidence of caesarean section, being cognisant of employing methods for monitoring, such as intermittent or wireless monitoring, as well as the supportive care required to advocate for physiological practises, may have a larger impact on reducing index caesarean rates.

### Barriers to use of wireless monitoring

In this survey, the next most common type of monitoring was ‘multiple’ forms of monitoring, which usually included failure of wireless CTG and progression to fetal scalp electrodes and/or wired CTG monitoring. When we examined why multiple forms occurred, using free text responses provided by respondents, it was mainly due to inadequate preparation of wireless devices (e.g. battery ran out), equipment failure, or failure to maintain an adequate fetal heart rate trace. This may be indicative of hospital resources, such as staffing, time required for management of intermittent handheld monitoring or the wireless equipment itself. This is suggested by a survey of nurses and midwives in a USA hospital, and by recent Australian research, where hospital staff report large portions of time on duty could be taken up ‘fiddling’ with wireless devices [6, 20], which is combined with limited availability of wireless devices available across all hospital types for women to use [8]. Their survey also indicated that hospital managers reported that devices tended to be reserved for women who indicated that they planned to be mobile in labour and not have an epidural, requiring women to initiate and manage their labour plans and monitoring in advance, rather than an embedded culture of supporting physiology for all women. This also has implications for midwifery skills and training for the professions [21], and points to further systemic issues raised by Lame and colleagues [7].

In other quantitative results from this study, which are reported in a separate paper [18], 70% of women who experienced wired CTG indicated that they would not choose wired CTG monitoring again. Even women who experienced wireless CTG/telemetry monitoring were more likely to say they either would not choose it again or were unsure if they would. In our study, almost no women had non-invasive adhesive monitoring (NIFECG), and intermittent handheld monitoring was mostly used only in birth centres and homebirth settings, raising the issue of equity of access to these models of care for women in Australia. Just under 50% of both multiparous and primiparous women experienced handheld monitoring in birth centres and 95% experienced it in homebirth settings. Qualitative results from this survey suggest that women would not choose wired CTG again due to discomfort and mobility. They found that the monitoring restricted their movement in labour, which was problematic for their sense of choice and control [16].

### Resource availability and workplace culture

Resource availability is central to routine provision of telemetry and handheld monitoring, and the cost of the equipment may be a factor for many hospitals. However, for private hospitals, where we saw the highest rates of wired CTG monitoring and where more resources may be available, investment in less invasive forms of monitoring, or models of care, which may be supportive of greater woman-centred approaches to birth, should be prioritised. However, issues with staffing levels and time availability, has been suggested by Australian research [6], and USA based research [20] as a major barrier to providing more resource intensive and woman-centred models of care. Financial investment includes providing telemetry monitoring, or newer non-invasive fetal ECG monitoring and prioritising having it available for each room. However, equipment alone is unlikely to improve women’s experiences. Staffing ratios and investment in training in woman-centred and continuity models of care along with support for physiological birth practises also need to be prioritised for women receiving any form of monitoring. The promotion of physiological birth and woman-centred models of care have repeatedly demonstrated significant benefits for women, midwives and hospitals [2, 6, 22], including reduction in costs for hospitals and government [23–28].

Workplace culture has also been implicated in a mixed methods study conducted by Watson et al., in 2022 in the UK study regarding type of monitoring used. The study suggests that birth centres and continuity models of care offered wireless and intermittent monitoring as routine, with midwives suggesting its use is ‘embedded’ in the workplace culture. Women tended to be more upright and mobile in labour and more likely to birth in upright and/or forward positions [13]. However more obstetric led models of care had an increased reliance on wired CTG monitoring, and greater likelihood of birth in recumbent or lithotomy positions. The study described midwives’ challenges in using wireless CTG monitoring, due to time required as well as training and experience. They also described their difficulty in supporting physiological labour and birth in units that were busy and catered to complex pregnancies, often reporting that they did not think to offer wireless CTG monitoring to women [13].

Resource implications are greater than the mere provision of monitoring devices, and extends to investment in research and development of well designed, less invasive wireless monitoring devices, as well as ongoing training for midwives and obstetric staff in the use of handheld monitoring. Perhaps the most important investment however, is in expanding woman-centred continuity of midwifery care models and training for midwives, so that care providers are able to promote physiological birth practices by having the capacity to spend time in “being with woman and not with machine”, as suggested by Fox and colleagues [6].

### Autonomy and freedom of movement in labour

It is essential to consider women’s capacity for autonomy and bodily freedom when investigating fetal heart rate monitoring in labour. In this study, women who experienced wired CTG monitoring were more likely to use pharmacological pain management such as an epidural, nitrous oxide gas and opioids, and were the least likely to report using ‘supportive care’ techniques for pain management. Women who used telemetry and handheld monitoring were more likely to use non-pharmacological techniques such as movement, breathing, massage, acupressure and supportive care from partners or care providers. This is a unique finding from this study, showing the relationship of type of monitoring and women’s use of pain management strategies, which are clearly impacted. Women’s capacity to use supportive care and non-pharmacological techniques, assisted by freedom of movement, appears to be directly related to type of monitoring in this study. This finding supports the literature on women’s, partners’ and midwives’ satisfaction with use of non-pharmacological pain management in labour and the impact of monitoring on freedom of movement and bodily autonomy [6, 12, 16, 29].

In this study, primiparous women, were more likely to receive wired CTG monitoring, and less likely to give birth in free standing birth centres or to have a home birth. National reporting data indicate that primiparous women are increasingly more likely to have medical interventions, including induction of labour, with no medical indication, and to experience augmentation in labour [3]. These women need to be given support with continuity models of care that are low intervention and favour intermittent handheld monitoring to utilise physiological practices to support normal birth. Australian women report wanting greater access to continuity models of care [30], which according to the results from this study, suggest women who were in continuity of care programs (birth centre and homebirth) were more likely to receive supportive care in the form of physical and emotional support, less invasive forms of fetal heart rate monitoring and increased likelihood of normal physiological birth, particularly for primiparous women. Other research by Homer (2016) and Tracy (2014) supports the benefit of continuity of care models for improved maternal outcomes and higher use of intermittent handheld monitoring, in particular for primiparas [31, 32]. Reducing rates of caesarean section in the index pregnancy, would greatly contribute to increased rates of normal births in subsequent births.

### The implications of induction and augmentation of labour

According to the most recent Australian Mothers and Babies report [3], over 35% of labours are induced, which is a common indication for continuous fetal monitoring in most guidelines [1, 2, 33–35]. The report states that only 41% of women in Australia overall commence labour spontaneously, and of these 28% will experience augmentation of labour, which is also indication for continuous fetal monitoring, and is more likely among primiparous women [3]. In previous research, women were asked retrospectively what they would have liked to have known before their first childbirth [36, 37]. Most commonly women cited the process involved in induction of labour, in particular the monitoring required, which was felt to have not been adequately explained to them. This is an important consideration, as rates of intervention are increasing rapidly over time [3]. Where women experience wired monitoring, they reported being restricted in their freedom of movement in labour, use of water for pain management and other non-pharmacological support, which requires upright and mobile positions to support physiological birth as has been reported in the literature [8, 38]. Type of monitoring should therefore be considered when discussing induction of labour with women, as this has been identified as a major factor impacting comfort and mobility.

### Information about monitoring for women

Information about monitoring should be explained fully, on multiple occasions and early, so that women are able to gain an in-depth understanding of the risks and benefits of fetal monitoring and give informed consent. According to the NICE guidelines, women should have fetal monitoring options discussed with them by all care providers, including antenatal visits and education, which describes the risks and benefits in an evidence-based framework [39]. This is supported by state and national guidelines in Australia [2, 33, 34, 40–43]. Women in this study who had a normal vaginal birth were more likely to have experienced handheld or wireless monitoring or fetal scalp electrode monitoring, and women who had a caesarean section or instrumental vaginal birth were more likely to have had wired monitoring, and more likely to have used epidural analgesia for pain management. Even when we controlled for epidural use, women who had wired CTG monitoring were still more likely to have a caesarean section. While we cannot state a causative effect, or the direction of relationship, it is clear from the literature [8, 16, 38], and from results of this study that the relationship of lack of freedom of movement has an impact of women’s experience of pain, and their agency to manage their pain with non-pharmacological techniques, particularly for first time mothers.

Collectively, these findings suggest that the burden remains on women to understand the impact of monitoring and to have planned for, and articulated their wishes prior to birth. This suggests that routine care is often not woman-centred or embedded in a culture of encouragement and support for handheld, or wireless monitoring, especially for primiparous women. Women in our study, reported in the qualitative results [16], as well as in other qualitative studies by Coddington et al., [44] and Watson [13], that they felt they were being ‘tethered’ or ‘strapped down’ to the bed when using wired CTG monitoring, which is deeply concerning. A recent survey of Australian women by Keedle et al., (2022), reported that more than 50% of women felt traumatised by their birth experience [45]. It is worth exploring info-graphic decision aid options for the presentation of information about monitoring options. This information should accompany any discussion of induction of labour or interventions due to risk status, which require continuous fetal monitoring in labour.

### Perception of benefits and harms

Finally, when we asked women about their perception of whether monitoring had a beneficial or negative impact on themselves or their babies, women who had wired CTG monitoring indicated that they felt there was no beneficial effect of monitoring for themselves, and that it had a negative impact on their labour. However, while women overall neither agreed nor disagreed about benefit for their baby, they did not think it had a negative impact. The current narrative of birth in Australia and many countries internationally, is one of women’s sacrifice of bodily autonomy and freedom for the perceived benefit of the baby [46]. Wired CTG monitoring is associated with increased interventions, less satisfaction for women and midwives, and reduced perceived comfort and benefit for women. This approach to increasing interventions is one that requires re-orientation of women’s position, if we are to achieve a humanised approach to birth [46], with the embodiment of philosophical approaches by midwives leading the change to supportive humanised approaches to childbirth for all women [47].

### Strengths and limitations

This national survey examined valid responses from 861 women from all states and territories in Australia. A limiting feature of survey research is that the method of distribution via online platforms may result in populations that do not represent the broader population more generally. We used paid advertisements to create a broad reach that was beyond our own networks to mitigate selection bias. Our survey respondents were more likely to represent women who had higher education and income levels and were two years older than the national average of 31.1 years. They were also more likely to be born in Australia than the average of 65.6% [3], and there was lower representation of women who identified as Aboriginal and Torres Strait Islander (3.3%) compared to the Australian average of 5.1%. There was also lower representation from women in metropolitan areas than the average of 73.9% according to the AIHW Mothers and Babies Report [3]. This may have influenced findings which are less representative of this population of women, in particular women from culturally, ethnically and linguistically diverse (CEALD) backgrounds or Aboriginal and Torres Strait Islander women, who are known to experience discrimination and lack of cultural safety in mainstream maternity care settings [48–50]. However, research into the experiences of these women is a priority area [51, 52]. Future research could explore priority groups’ experiences of labour, in language, and the use of fetal monitoring technologies to capture the views of more diverse populations.

Another limitation of this study was that data for induction of labour was not able to be analysed, nor accounted for in statistical analysis as a potential confounder. While induction of labour is a common indication for continuous electronic fetal monitoring, this survey was focused on women’s experiences of monitoring regardless of labour onset. We have noted the issue in the survey design for future studies. Results should be interpreted in light of these limitations.

## Conclusion

This study has substantial and multifactorial implications for research, hospital management and resources, models of care, guideline and policy implementation as well as practice. This includes evaluating the implementation of less invasive and more humane forms of fetal monitoring in childbirth, expansion of access to continuity models of care, particularly for primiparous women, and providing easy to understand evidence-based information for all women. Hospitals need to address workflow and cultural issues, and enhancing staffing ratios to support the use of less invasive forms of fetal monitoring. If we are to honestly contribute to the humanisation of childbirth for all women, the implementation of evidence-based practices that support woman-centred models of care must be reinforced at every level of care.

## Data Availability

The datasets used and/or analysed during the current study are available from the corresponding author on reasonable request.

## Abbreviations

WOMB Study: Women’s experiences Of Monitoring Baby.
EFM: Electronic fetal monitoring.
CTG: Cardiotocography.
NIFECG: Non-invasive fetal electrocardiography.
Multiple monitoring: Where two or more main forms of monitoring were utilised, not including a routine CTG trace as part of the admission process.
CS: Caesarean section.
emCS: Emergency caesarean section.
elCS: Elective caesarean section.
CEALD: Culturally, ethnically and linguistically diverse.
AMU/FMU: Midwifery-led models of care at ‘Birth centres’, includes freestanding and alongside units. These are known internationally as Alongside Midwifery Units (AMU), which are located inside the hospital building, and Freestanding Midwifery Units (FMU), which are separate from the hospital building.

## Declarations

### Inclusivity statement

We have chosen to use the word ‘woman’ to describe respondents to the survey, and to reflect female physiology in childbirth. However, we respect gender diversity and acknowledge that not all people who are pregnant or giving birth identify as women. This paper reflects the views of respondents as representative of a cohort giving birth in Australia.
